# RNA pathogenesis via Toll-like receptor-activated inflammation in expanded repeat neurodegenerative diseases

**DOI:** 10.3389/fnmol.2013.00025

**Published:** 2013-09-05

**Authors:** Robert I. Richards, Saumya E. Samaraweera, Clare L. van Eyk, Louise V. O’Keefe, Catherine M. Suter

**Affiliations:** ^1^Discipline of Genetics and Centre for Molecular Pathology, School of Molecular and Biomedical Science, The University of AdelaideAdelaide, SA, Australia; ^2^Victor Chang Cardiac Research InstituteDarlinghurst, NSW, Australia

**Keywords:** RNA pathogenesis, Toll-like receptor, innate inflammation, expanded repeat diseases, neuro-degeneration

## Abstract

Previously, we hypothesized that an RNA-based pathogenic pathway has a causal role in the dominantly inherited unstable expanded repeat neurodegenerative diseases. In support of this hypothesis we, and others, have characterized *rCAG.rCUG*_100_ repeat double-strand RNA (dsRNA) as a previously unidentified agent capable of causing pathogenesis in a *Drosophila* model of neurodegenerative disease. *Dicer*, *Toll*, and autophagy pathways have distinct roles in this *Drosophila *dsRNA pathology. *Dicer* dependence is accompanied by cleavage of *rCAG.rCUG*_100_ repeat dsRNA down to *r(CAG)*_7_ 21-mers. Among the “molecular hallmarks” of this pathway that have been identified in *Drosophila*, some [i.e., *r(CAG)*_7_ and elevated tumor necrosis factor] correlate with observations in affected people (e.g., Huntington’s disease and amyotrophic lateral sclerosis) or in related animal models (i.e., autophagy). The *Toll* pathway is activated in the presence of repeat-containing dsRNA and toxicity is also dependent on this pathway. How might the endogenously expressed dsRNA mediate *Toll*-dependent toxicity in neuronal cells? Endogenous RNAs are normally shielded from *Toll* pathway activation as part of the mechanism to distinguish “self” from “non-self” RNAs. This typically involves post-transcriptional modification of the RNA. Therefore, it is likely that *rCAG.rCUG*_100_ repeat dsRNA has a characteristic property that interferes with or evades this normal mechanism of shielding. We predict that repeat expansion leads to an alteration in RNA structure and/or form that perturbs RNA modification, causing the unshielded repeat RNA (in the form of its *Dicer*-cleaved products) to be recognized by *Toll*-like receptors (TLRs), with consequent activation of the *Toll* pathway leading to loss of cell function and then ultimately cell death. We hypothesize that the proximal cause of expanded repeat neurodegenerative diseases is the TLR recognition (and resultant innate inflammatory response) of repeat RNA as “non-self” due to their paucity of “self” modification.

## INTRODUCTION

Since the first discovery of trinucleotide repeat expansion as the basis for many important human genetic diseases (Kremer et al., 1991; La Spada et al., 1991; Yu et al., 1991; Richards and Sutherland, 1992), there has been a vast amount of research in this area (*PubMed *search “trinucleotide repeat disorders” gives >3,700 results). Much of this research is aimed at identifying the mechanism of pathogenesis underlying diseases caused by this form of mutation. Individual diseases can follow either dominant or recessive mode of inheritance indicating distinct pathogenic pathways. Repeat sequences that are expanded in copy number are the basis for ~20 dominantly inherited neurodegenerative diseases, including Huntington’s disease (HD). Despite some of the responsible genes being identified as long as 20 years ago, the identity and nature of the disease-causing pathogenic pathway remains a gap in knowledge for these diseases, i.e., no definitive molecular pathway from the mutation to the clinical symptoms has yet been identified. For at-risk individuals in families affected with dominantly inherited late-onset neurodegenerative diseases due to expanded repeats, the majority opt not to have the definitive pre-symptomatic diagnostic test. Their preference is to live with the uncertainty of not knowing, than the certainty of getting the disease, as no treatments are yet available. Therefore, determining the pathogenic pathway and identifying therapeutic targets for intervention is an urgent priority for reducing the impact of these devastating diseases. This understanding is essential for rational approaches to delay onset, slow progression, or ultimately effect cure.

## MOLECULAR PATHWAY FROM REPEAT EXPANSION TO DISEASE

There are common properties exhibited by the various repeat expansions that give rise to human disease. The vast majority of these diseases originate from an existing repeat sequence that exhibits copy number variation in the human population. In each case, the disease alleles arise when copy number increases beyond a critical threshold. The repeat composition varies, but most are trinucleotide repeats. In some diseases, the repeat expands to the point where gene expression at the expanded repeat locus is either substantially reduced or lost altogether, resulting in loss-of-function of the repeat-harboring gene. Typically such diseases are inherited in a recessive manner. Many repeat loci, however, give rise to dominantly inherited diseases in a manner that is not gene-dose dependent (i.e., two mutant alleles are no worse, and may be even better than one – see Carroll et al., 2013). This suggests that gain-of-function is the mechanism rather than haploinsufficiency. Repeat copy number in many cases is a major determinant of age at onset of clinical symptoms (referred to as “anticipation”) indicating that the repeat itself is a rate-limiting determinant of the pathogenic pathway. However, since pathology typically involves cell death and there are many ways in which cells die, identification of the disease-causing “toxic agent” has been problematic.

## IS THERE A COMMON PATHOGENIC AGENT?

**FIGURE 1:**
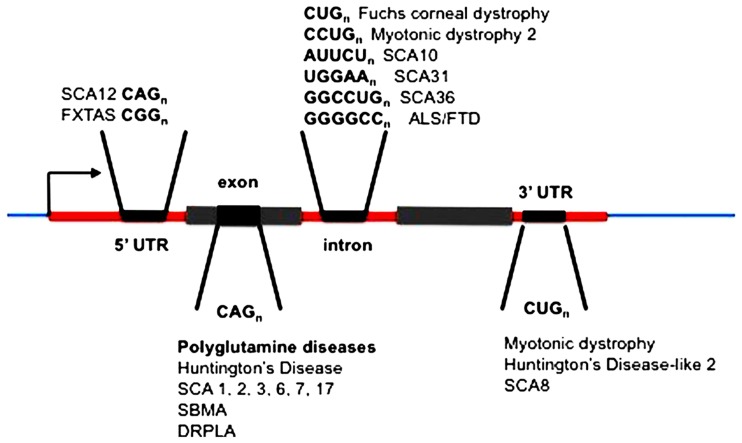
**Location of expanded repeats in disease genes.** SCA, spinocerebellar ataxia (multiple loci numbered); FXTAS, fragile X tremor ataxia syndrome; ALS, amyotrophic lateral sclerosis; FTLD, frontotemporal lobar dementia; SBMA, spinobulbar muscular atrophy; DRPLA, dentatorubral-pallidoluysian atrophy.

The unstable expanded repeat diseases (**Figure 1**) typically manifest as neurodegenerative and/or muscular diseases, some with a high degree of clinical overlap, despite affecting distinct proteins and unrelated loci. Where expanded repeats are translated, they generally code for polyglutamine; however, the proteins in which they are located are all unrelated in the remainder of their amino-acid sequence. Therefore, much attention has been focused on expanded polyglutamine as the common basis of pathology (McLeod et al., 2005; van Eyk et al., 2011, 2012). Some of these diseases, however, have repeat expansions located within untranslated RNAs and/or arise from repeat sequences that cannot encode polyglutamine (**Figure 1**; Richards, 2001; La Spada and Taylor, 2010). Despite these significant differences in the location of the repeat in this family of diseases they exhibit overlapping symptoms resulting from neuronal loss of function and/or neurodegeneration. In addition, in most cases the polyglutamine and “untranslated” diseases have similar disease allele copy number repeat thresholds (*HD *CAG > 36, *SCA17 *CAG > 47, *FXTAS *CGG > 55, *HDL2 *CUG > 44, *DM1 *CUG > 50, *SCA12 *CAG > 66). This suggests that there may be a common pathogenic agent or agents in the translated and untranslated repeat diseases.

## RNA MAY BE PATHOGENIC IN TRANSLATED REPEAT DISEASES

While there is growing consensus that RNA plays a causal role in “non-coding repeat expansion disorders,” its contribution when the repeat is located in coding regions (specifically polyglutamine disorders) is more controversial (Fiszer and Krzyzosiak, 2013). Yet even here there is evidence that RNA is key. For example, intermediate copy number CAG alleles of *SCA2 *that are below the threshold required to encode aggregate forming polyglutamine, increase the risk of amyotrophic lateral sclerosis (ALS; Elden et al., 2010). Furthermore, interruption of CAG repeat with CAA dramatically mitigates polyglutamine toxicity in a *Drosophila *model of *SCA3* (Li et al., 2008).

It is possible that multiple pathways (at least one of which is RNA mediated) contribute to progression of expanded repeat neurodegenerative diseases. In support of this possibility, ALS and *SCA7 *both appear to involve two cell types (nerve cells and glial cells; Furrer et al., 2011; Polymenidou and Cleveland, 2011). Astrocytes and glial cells have both been shown to affect their neighboring neurons in individuals with repeat expansions, leading Ilieva et al. (2009) to hypothesize that the onset of the disease is determined in the nerve cell, and the progression of the disease determined in adjacent glial(-like) cells. But importantly, there is consistent evidence implicating RNA as of principle importance as the originating causal event that initiates pathology.

## MECHANISMS OF RNA-INITIATED PATHOLOGY

**FIGURE 2:**
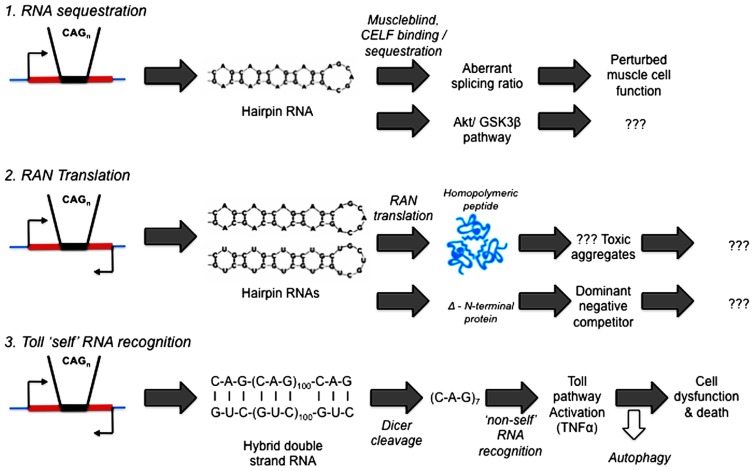
**Competing hypotheses of expanded repeat disease pathogenic pathways involving RNA.** (1) RNA sequestration – via alternative splicing (Mankodi et al., 2002; Ranum and Day, 2004) or Akt/GSK3β pathway (van Eyk et al., 2011; Jones et al., 2012; Lawlor et al., 2012). (2) RAN (repeat associated non-AUG) Translation (Zu et al., 2011; Ash et al., 2013; Mori et al., 2013; Todd et al., 2013). (3) *Toll* “self” RNA recognition (Lawlor et al., 2011; Yu et al., 2011; Samaraweera et al., 2013).

What precedents and potential mechanisms are there for RNA to initiate pathogenesis in human diseases? See **Figure 2**.

### SINGLE-STRANDED RNA TOXICITY

Precedence for expanded repeat RNA being a disease-causing entity in its own right first came from the *DM1 *and *DM2 *repeat expansions that both give rise to myotonic dystrophy (Ranum and Day, 2004). The repeat expansions in these diseases are similar, but importantly, not identical (CUG vs. CCUG) and are located in untranslated regions (3′UTR or intron) of two otherwise unrelated genes (*DMPK *and *ZNF9*). In muscle cells, RNAs from expanded alleles of either repeat are able to bind and sequester alternative splicing factors (muscleblind and CUG-BP) and in so doing, perturb the splicing pathways of proteins for which alternative splicing is a necessary step for their complete range of functions (Mankodi et al., 2002; Ranum and Day, 2004). It is now generally accepted that RNA is the common pathogenic agent in these diseases most likely through its impact on alternative splicing, although this has recently been challenged with evidence that GSK3β mediates at least some aspects of the RNA-based pathology in myotonic dystrophy (Jones et al., 2012) and in a *Drosophila *model (van Eyk et al., 2011).

Evidence for a more widespread role for RNA in neurodegenerative diseases has been steadily accumulating. *SCA31 *and *SCA36 *are due to large expansions of *de novo *5 bp TGGAA repeat and an existing 6 bp GGCCTG repeat, respectively – both located within introns of different genes (Sato et al., 2009; Kobayashi et al., 2011). An expanded GGGGCC repeat has recently been found to cause a substantial proportion of cases of ALS and frontotemporal lobar dementia (FTLD; DeJesus-Hernandez et al., 2011; Renton et al., 2011). As indicated by others (Orr, 2011) “The location of this repeat within an intron of the *C9ORF72 *gene along with some evidence for alternative splicing of *C9ORF72 *transcripts brings in to play a prominent aspect of non-coding repeat expansion disorders – the role of RNA metabolism in pathogenesis.”

### REPEAT ASSOCIATED NON-AUG TRANSLATION

The hairpin structure of expanded repeat RNA is such that it can enable the initiation of translation in the absence of the normal requirement of an AUG start codon (Zu et al., 2011). Although this mechanism involves conversion of the RNA into peptides, thereby rendering the RNA no longer “untranslated,” the phenomenon can occur to RNA sequences that do not normally appear in protein-coding sequences, i.e., RNA from introns or 5′ or 3′ untranslated regions of mRNAs. The resultant translated polypeptides can initiate from within the repeat sequence and in any reading frame, therefore, a single strand containing repeat RNA sequence can encode three different polypeptide sequences. Since expanded repeat sequences are typically located in regions of bi-directional transcription (Batra et al., 2010), the resultant transcripts from both strands potentially enable the production of six different peptide sequences, any of which may be toxic to the cell. Such polypeptides have now been detected in pathology specimens from individuals affected with a number of different expanded repeat diseases including DM1, fragile X syndrome (FRAXA) and ALS/FTLD (Zu et al., 2011; Ash et al., 2013; Mori et al., 2013; Todd et al., 2013). Of particular note, two recent publications (Ash et al., 2013; Mori et al., 2013) have identified repeat associated non-AUG (RAN)-translation of the GGGGCC expanded repeats that cause ALS/FTLD into polypeptides that also form aggregates in affected tissues. However, these aggregates are confined to nerve cells and are absent from adjacent glial cells that are also involved in the pathology. On the other hand, the absence of visible aggregates does not prove the absence of toxic peptides.

These RAN translation results have suggested that an aggregate polypeptide analogous to polyglutamine could be neurotoxic in diseases where the causative repeat expansion cannot encode polyglutamine. However, this is doubtful in the cases of ALS and FTLD because of the observations that mutations in either of two RNA-binding proteins, FUS and TDP-43, can also cause disease (Rutherford et al., 2008; Van Langenhove et al., 2010). In individuals affected due to these mutations, no such expanded polyGly-Pro polypeptide is evident, therefore while polyGly-Pro may lead to subtle differences in pathology (Ash et al., 2013; Mori et al., 2013), it appears to play a modifying role at most.

Furthermore, inhibition of an RNA lariat debranching enzyme has recently been shown to suppress TDP-43 toxicity in ALS disease models (Armakola et al., 2012). These observations reinforce the view that RNA has a central role to play in this disease. While the role of such polypeptides in disease pathogenesis is unclear, for example, whether their aggregation may actually be protective rather than pernicious, they are a curious set of products driven by the unusual structure of expanded repeat RNAs. One possibility is that rather than the homopolymeric polypeptides themselves being toxic, the initiation of translation within the repeats could give rise to *N*-terminal truncated proteins devoid of upstream functional domains that could then act as dominant negative competitors for the full-length functionally intact proteins.

### DOUBLE-STRANDED EXPANDED REPEAT RNA IS PATHOGENIC

*Drosophila *models of expanded repeat diseases have been described that specifically investigate the intrinsic toxicity of both translated and untranslated expanded repeat sequences (Lawlor et al., 2011; van Eyk et al., 2011, 2012; Samaraweera et al., 2013). In one study (Lawlor et al., 2011), a single line of *Drosophila *expressing untranslated CAG was identified with a marked degenerative phenotype (whereas multiple other random insertion lines of the same transgene had no such phenotype). Upon detailed characterization, this degenerative phenotype line was found to have the repeat transgene inserted into an endogenous gene (*cheerio*) in the opposite orientation to normal transcription. Transcripts containing expanded repeats would, therefore, originate from both strands via bi-directional transcription. This finding coincided with numerous reports in the literature that expanded repeat disease loci are typically transcribed from both DNA strands (see Batra et al., 2010). Therefore, this *Drosophila *line mimicked a previously uncharacterized property of these disease genes. Bi-directional transcription was subsequently modeled in a controlled manner by co-expression from two different transgenes of expanded *rCAG*_~100_ together with *rCUG*_~100_ [giving rise to *rCAG.rCUG*_~100_ or double-strand RNA (dsRNA)] to produce repeat-containing dsRNA (Lawlor et al., 2011). Flies expressing dsRNA showed *Dicer*-dependent toxicity. Additionally dsRNA expression throughout the nervous system caused an age-dependent neurodegenerative phenotype. An abundance of *r(CAG)*_7_ also implicated specific *Dicer *processing of the *rCAG.rCUG*_~100_ dsRNA as a pathogenic pathway in this model (Lawlor et al., 2011). Similar findings have also been reported in an independent *Drosophila *model (Yu et al., 2011). There are, with all animal models, caveats. In order to manifest a phenotype in the time frame of laboratory experiments, these *Drosophila *(and other animal) models employ copy numbers well in excess of those that cause pathology (after several decades) in some of these diseases. This is thought to be due to an inverse relationship between repeat copy number and age-at-onset, the basis of which could be somatic repeat instability over time (see **Figure 2** in Richards, 2001 and Swami et al., 2009). Furthermore, the level of expression of the repeat RNAs required to give an early phenotype in animal models (Lawlor et al., 2011; Yu et al., 2011) may be well in excess of that of the endogenous human disease gene. Importantly, however, examination of HD patient samples (Bañez-Coronel et al., 2012) revealed the presence of the same *r(CAG)*_7_ cleavage product seen in the *Drosophila *models, providing evidence in support of the activity of this pathway in HD pathogenesis.

In an effort to identify further components of expanded repeat RNA pathogenesis in *Drosophila*, microarray analyses of *Drosophila *expressing *rCAG.rCUG*_~__100_ dsRNA have been undertaken (Samaraweera et al., 2013). Changes in transcription profiles revealed candidate pathways for mediating the resultant pathogenesis. Alterations in transcripts common to several pathways were detected, including components of inflammation and innate immunity. Hallmarks of immune activation, including elevated plasma tumor necrosis factor (TNF), appear prior to clinical symptoms of dominantly inherited expanded repeat human diseases (Moreau et al., 2005; Björkqvist et al., 2008). Therefore, the *Drosophila *model expressing *rCAG.rCUG*_~__100_ dsRNA was utilized to test two key elements of immune activation – the *Toll *and autophagy pathways for their contribution to expanded repeat RNA pathogenesis. *Toll *signaling pathway was identified as essential for dsRNA pathogenesis and autophagy was found to reduce toxicity in this model (Samaraweera et al., 2013). Furthermore, multiple reports implicate glial cells in the pathology of expanded repeat diseases. Neurons are dependent upon glial cell function that includes the destruction and removal of the carcasses of dead neurons. The *rCAG.rCUG*_~__100_ dsRNA was found to impact nerve cell function even when exclusively expressed in glial cells (Samaraweera et al., 2013), providing evidence that dsRNA pathology in *Drosophila *is, like the human expanded repeat diseases, non-cell autonomous (Ilieva et al., 2009; Furrer et al., 2011).

The requirement for *Toll *signaling pathway in this *Drosophila *model is intriguing.* Toll*-like receptors (TLRs) function in normal biology to protect an organism from infection by viruses and bacteria. They recognize foreign pathogen molecules including DNA and RNA through specific receptors (such as endosomal *TLR3*) and can distinguish these nucleic acids (as “non-self”) from the endogenous nucleic acids (“self”). Therefore, while the *rCAG.rCUG*_~_100 dsRNA is being expressed endogenously in this *Drosophila *model, it is being recognized by the *Toll* signaling pathway as foreign or “non-self” – a recognition that then activates innate inflammatory regulatory pathways, ultimately leading to cell death.

## PATHOGENIC MUTATIONS IN PROTEINS THAT FUNCTIONALLY INTERACT WITH RNA

While it can be difficult to ascribe specific functions to RNA in pathogenic pathways, there are some noteworthy instances of disease-causing mutations in proteins that functionally interact with RNAs. By implication, the RNAs that these proteins normally act upon are, therefore, likely contributors to and/or mediators of the relevant pathogenic process.

### RNA-BINDING MOTIFS – THE RNAS THAT HAVE THEM AND THE PROTEINS THAT RECOGNIZE THEM

Recent discoveries regarding the importance of RNA–protein recognition in disease pathogenesis have led to a renewed interest in the role that these interactions play in biological processes. While they have long been recognized as key regulators of gene expression, only a small fraction have been functionally characterized. A recent compendium of RNA-binding motifs (Ray et al., 2013) highlighted both the significance and scope of these interactions. The human genome encodes at least 400 known or predicted RNA-binding proteins with a diverse array of RNA sequence-binding motifs. Indeed the number of such human RNA-binding proteins appears to be much higher than this, with 860 identified in HeLa cells alone (Castello et al., 2012). The scope and specificity of RNA recognition is determined both by the number and variety of RNA-binding proteins and by the number and variety of RNA-sequence motifs that they bind.

### FRAGILE X SYNDROME IS DUE TO LOSS OF RNA-BINDING PROTEIN FUNCTION

Fragile X syndrome is a striking example of the role of an RNA-binding protein in human disease. FRAXA is due to the expanded CGG repeat that is responsible for the *FRAXA* rare, folate-sensitive chromosomal fragile site (Kremer et al., 1991), located in the 5′UTR of the *FMR1* gene (Verkerk et al., 1991). Expansion of the repeat beyond ~230 copies results in inactivation of the gene and consequent loss of encoded FMRP (fragile X mental retardation protein) function (Pieretti et al., 1991). The FMRP is an RNA-binding protein with KH- and RGG-binding motifs (Ashley et al., 1993). The loss of function of this protein is responsible for the clinical symptoms as rare cases of point mutation or deletion of the *FMR1* gene have similar clinical symptoms. Indeed one of these pathogenic point mutations is at a highly conserved amino acid in a KH domain of FMRP highlighting the significance of the role of RNA interaction in FMRP function (De Boulle et al., 1993). The FMRP has an impact on the translation of the mRNAs with which it interacts (Darnell et al., 2001) and, therefore, its absence leads to the dysregulation of the translation of these specific mRNAs. This is thought to be the proximal cause of the symptoms of FRAXA.

### THE INTRIGUING PATHOGENESIS OF AICARDI–GOUTIÈRES SYNDROME

**FIGURE 3:**
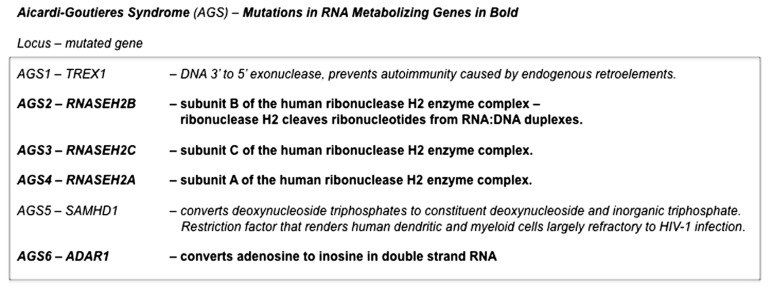
**Pathogenic mutations in Aicardi–Goutières syndrome**. Mutations in genes in at least six distinct loci are able give rise to the constellation of symptoms that defines Aicardi–Goutières syndrome. Four of these (AGS2, AGS3, AGS4, and AGS6) are in genes that encode RNA-metabolizing proteins. The remaining two that have been identified (AGS1 and AGS5) are also in enzymes that have roles in nucleic acid metabolism. Deficiencies in any one of these enzymes are thought to result in the accumulation of endogenous nucleic acids that are sensed as “non-self” by *Toll*-like receptors, that in turn activate innate inflammatory pathways (Crow and Rehwinkel, 2009).

Aicardi–Goutières syndrome (AGS) is a genetically heterogeneous disorder that is due (at least in a substantial proportion of cases) to the mutation of various nucleic acid-metabolizing enzymes, including various subunits of ribonuclease H2 or the RNA-editing enzyme ADAR1 (see Crow and Rehwinkel, 2009 and OMIM #225750). AGS is characterized, in its more severe forms, by severe neurological dysfunction in infancy that includes progressive microcephaly, spasticity, dystonic posturing, profound psychomotor retardation, and often death in early childhood (OMIM #225750) (**Figure 3**). In its milder forms, these neurological symptoms are diminished or even absent, but peripheral symptoms outside the nervous system are common to the phenotypic spectrum and include thrombocytopenia, hepatosplenomegaly, and elevated hepatic transaminases along with intermittent fever. Chilblains are also a typical feature. Together these symptoms demonstrate phenotypic overlap both with systemic lupus erythematosus and with the sequelae of congenital infection (Crow and Rehwinkel, 2009). The disease, therefore, appears to be due to defects in the processes that remove and/or modify endogenous nucleic acids. These endogenous unmodified nucleic acids then accumulate and are sensed as “non-self” by TLRs, that, in turn, activate innate inflammatory regulatory pathways. This bears a striking resemblance to mechanisms we have identified as responsible for dsRNA pathogenesis in the *Drosophila *model of expanded repeat neurodegenerative diseases.

## HYPOTHESIS

### EXPANDED REPEAT RNAS AS PATHOGENIC AGENTS BY *TOLL* “SELF” RNA RECOGNITION

**FIGURE 4:**
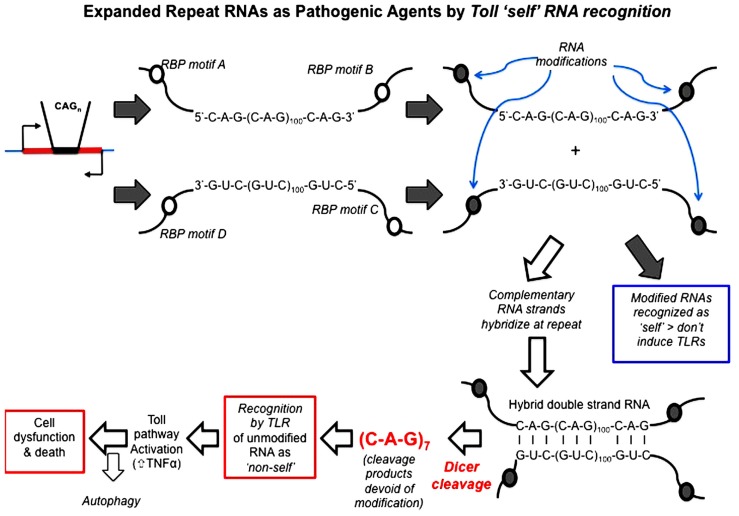
**Hypothesis: expanded repeat neurodegenerative diseases are caused by the TLR recognition (and resultant innate inflammatory response) of repeat RNA as “non-self” due to their paucity of “self” modification that is exposed upon Dicer processing of double-strand RNA.** Open circles represent sequence motifs for RNA modifying proteins; filled circles represent the modification of RNA at these specific sequence motifs (e.g., by methylation or A > I editing). Dicer is required for pathology in the *Drosophila *model and cleaves long high copy number repeat RNA down to 21mers [mainly *r(CAG)*_7_ mers; Lawlor et al., 2011]. These *r(CAG)*_7_ mers are, therefore, unmodified and recognized by TLRs as “non-self.” *Toll*-like receptor pathways (most probably the endosomal TLR3 receptor) are required for pathology (Samaraweera et al., 2013), through activation of the innate inflammatory pathway. Autophagy reduces pathology, possibly by metabolizing *r(CAG)*_7_ mers.

Repeat RNA sequences represent a pivotal point of potential weakness in processes that utilize RNA–protein recognition, as the repeat RNA sequence will harbor either a paucity or excess of sequence-binding motifs. Expansion of repeat RNA sequences, therefore, clearly has the potential to give rise to too much or too little of an interaction that is a rate-limiting factor in a crucial biological process. RNA modification is one process that is sequence motif-dependent and known to be key to the distinction between “self” and “non-self” by components of the innate immune pathways. Indeed, it has been shown that exogenous “non-self” RNAs require *in vitro* modification in order to escape innate immune recognition and activation when transferred *in vivo* (Warren et al., 2007; Pan, 2013). The exposure of the innate immune activators to unmodified nucleic acids, including RNA, appears to be the proximal cause of AGS. We, therefore, hypothesize that this provides a clear molecular mechanism for the ability of expanded repeat RNA sequences, through their paucity of RNA modification, to initiate pathogenesis in the dominantly inherited, expanded repeat neurodegenerative diseases (**Figure 4**).

## ACTIVITY OF *TOLL* “SELF” RNA RECOGNITION IN NEURODEGENERATIVE DISEASES

Double strand expanded repeat RNA pathology has been modeled in *Drosophila*. What evidence is there that this pathway of TLR recognition of expanded repeat RNA and subsequent activation of the innate inflammatory cascade is active in the human dominantly inherited neurodegenerative diseases due to expansion of repeat sequences?

One of the key steps in dsRNA pathology is the generation of *r(CAG)*_7_ 21mers from the much greater copy number double strand repeat RNA by *Dicer*. This *r(CAG)*_7_ 21mer has been identified in the brain RNA of individuals affected with HD (Bañez-Coronel et al., 2012). The activity of *Dicer* is crucial to the observed pathology in the *Drosophila *model (Lawlor et al., 2011) and, therefore, it would appear that this step is a likely proximal event in the observed phenotype. The appearance of *r(CAG)*_7_ 21mers in HD brain is therefore an important “molecular hallmark” of this pathway and a key indicator of its activity in the human disease. Another, albeit less direct, indicator of this pathway is seen in the increased activity of components of the innate inflammatory response mechanism in human diseases associated with expanded repeats. Elevated TNF is seen in the *Drosophila *model as one read-out of innate immune activation (Samaraweera et al., 2013) and both TNF and various interleukins (i.e., IL-4, IL-5, IL-6, IL-8, and IL-10) have been found to be elevated in people affected with the repeat expansion responsible for HD even before clinical manifestation of the disease (Björkqvist et al., 2008). Another indicator of innate immune activation in HD is the abnormal peripheral chemokine profile that has been observed in HD (Wild et al., 2011). Various reports indicate activation of innate adaptive immunity via TLR signaling in ALS (Casula et al., 2011; Sta et al., 2011) – a disease that has recently been found, at least in a proportion of instances, to be also due to an expanded repeat (DeJesus-Hernandez et al., 2011; Renton et al., 2011).

## CONCLUSION

A growing body of literature indicates a consistent association between innate immunity, neuroinflammation and neurodegeneration (Shastri et al., 2013). Where there are exogenous causes (e.g., trauma or infection), activation of the TLR pathway can be attributed to an external agent (e.g., bacterial lipopolysaccharide or viral RNA); however, a causal basis for this relationship has not been clear when there is an endogenous basis to the disease, e.g., expansion of a repeat sequence beyond a pathogenic threshold. Recognition by the *Toll* receptor pathway of expanded repeat RNA as “non-self” and consequent activation of the innate immune inflammatory cascade provides a mechanism and a common pathogenic pathway for the neurodegenerative diseases due to expanded repeats. This new understanding, once proven in the relevant human diseases, will provide new targets for intervention and ultimately, we hope, therapeutic targets for drugs to delay onset and/or alleviate disease progression.

## AUTHOR CONTRIBUTIONS

Robert I. Richards drafted the initial version of the manuscript, including the hypothesis, then edited in the additions and changes made by the other co-authors. Saumya E. Samaraweera and Clare L. van Eyk provided original unpublished data on which the manuscript and its hypothesis is based, as well as contributions to the development of the hypothesis, the text and figures. Louise V. O’Keefe contributed to the development of the hypothesis and additional text and revision of the manuscript. Catherine M. Suter contributed to information on RNA-binding proteins, the development of the hypothesis and content of the text and figures.

## Conflict of Interest Statement

The authors declare that the research was conducted in the absence of any commercial or financial relationships that could be construed as a potential conflict of interest.
